# Living Shorelines: Coastal Resilience with a Blue Carbon Benefit

**DOI:** 10.1371/journal.pone.0142595

**Published:** 2015-11-16

**Authors:** Jenny L. Davis, Carolyn A. Currin, Colleen O’Brien, Craig Raffenburg, Amanda Davis

**Affiliations:** 1 NOAA National Ocean Service Center for Coastal Fisheries & Habitat Research, Beaufort, North Carolina, United States of America; 2 Consolidated Safety Services, Fairfax, Virginia, United States of America; 3 Eckerd College, St. Petersburg, Florida, United States of America; 4 University of Miami, Rosenstiel School of Marine and Atmospheric Science, Miami, Florida, United States of America; 5 Massachusetts Division of Marine Fisheries, New Bedford, Massachusetts, United States of America; Fudan University, CHINA

## Abstract

Living shorelines are a type of estuarine shoreline erosion control that incorporates native vegetation and preserves native habitats. Because they provide the ecosystem services associated with natural coastal wetlands while also increasing shoreline resilience, living shorelines are part of the natural and hybrid infrastructure approach to coastal resiliency. Marshes created as living shorelines are typically narrow (< 30 m) fringing marshes with sandy substrates that are well flushed by tides. These characteristics distinguish living shorelines from the larger meadow marshes in which most of the current knowledge about created marshes was developed. The value of living shorelines for providing both erosion control and habitat for estuarine organisms has been documented but their capacity for carbon sequestration has not. We measured carbon sequestration rates in living shorelines and sandy transplanted *Spartina alterniflora* marshes in the Newport River Estuary, North Carolina. The marshes sampled here range in age from 12 to 38 years and represent a continuum of soil development. Carbon sequestration rates ranged from 58 to 283 g C m^-2^ yr^-1^ and decreased with marsh age. The pattern of lower sequestration rates in older marshes is hypothesized to be the result of a relative enrichment of labile organic matter in younger sites and illustrates the importance of choosing mature marshes for determination of long-term carbon sequestration potential. The data presented here are within the range of published carbon sequestration rates for *S*. *alterniflora* marshes and suggest that wide-scale use of the living shoreline approach to shoreline management may come with a substantial carbon benefit.

## Introduction

In recent years, the impacts of several major U.S. coastal storms including Hurricanes Katrina and Rita (2005) and Superstorm Sandy (2012) have highlighted the need for increased coastal resilience. Toward this goal, a number of recent works have emphasized the central role that both natural coastal ecosystems and nature-based infrastructure (hybrid approaches that include a combination of natural ecosystems and built components) can play in enhancing the resilience of our nation’s coastline to extreme weather events [1–3]. To date, living shorelines are one of the more commonly used examples of nature-based infrastructure [3].

By definition, living shorelines are a shoreline management option that involves the utilization of native marsh vegetation for the purpose of erosion control [4]. A living shoreline may consist of only vegetation; however, their design often includes grading, addition of fill prior to planting to achieve the correct elevation, and in many cases, an offshore structure composed of unconsolidated rock, natural fiber logs or created oyster reef that is intended to reduce wave energy [5–8]. The popularity of the living shoreline approach has grown in the past decade as multiple investigators have demonstrated the value of natural vegetated shorelines for wave attenuation and erosion control [1, 9, 10]. More recently, living shorelines were demonstrated to be more resilient to hurricane impacts than shorelines hardened with bulkheads [11]. Further, in contrast to traditional shoreline armoring devices like bulkheads, living shorelines preserve connectivity between upland and nearshore habitats and thus, provide many of the ecosystem services attributed to natural wetlands including pollution and nutrient removal and habitat provision [6, 7, 9, 12–14]. In the current study we investigate an additional potential service provided by living shorelines that has not yet been documented, their ability to sequester carbon.

Carbon sequestered (buried for long time periods) in tidal wetlands (termed blue carbon) has received increasing attention in recent years [15–18]. Tidal wetlands are characterized by high primary productivity and slow remineralization, and, therefore, tend to sequester carbon at much greater rates than terrestrial ecosystems [16]. These traits have fueled an interest in the creation and management of coastal wetlands for their value as carbon sinks [15, 19, 20], and led to the recent creation of a verified carbon standard for creation and restoration of coastal wetlands [21]. Introduction of this standard provides a mechanism for quantifying the emission offsets associated with wetland protection and creation/restoration activities, as well as, for valuing wetlands for trading in carbon markets. Development of this standard was aided by a number of studies which quantified rates of carbon sequestration in natural and restored tidal wetlands. These works have yielded rates that vary by an order of magnitude, presumably as a result of variability in plant community, climate and salinity [22, 23]. Much of what is currently known about carbon sequestration in created and restored wetlands is based on work in sites with expansive marsh platforms and large ratios of marsh interior area to edge. In contrast, living shorelines typically incorporate narrow fringing marshes, < 30 m in width. They tend to be well flushed and have sandy sediments with low organic matter content and, thus, represent a unique subset of intertidal marshes [7, 24]. As a result, it is unclear how well living shorelines are represented by the current literature on marsh carbon sequestration rates.

To the naked eye, created and restored marshes rapidly become indistinguishable from natural marshes. Vegetative characteristics like stem density, plant height and shallow (0–10 cm) belowground macro organic matter in a newly planted *Spartina alterniflora* marsh will typically mirror those of nearby natural marshes within two to three growing seasons [24, 25]. However, other characteristics are much slower to develop. The infaunal benthic community of created tidal salt marshes may take longer than a decade to develop to levels equivalent to those of natural marshes [26, 27]. Sediment biogeochemical processes appear to take even longer. Other investigators have suggested that multiple decades are required for the organic matter content of created marshes to reach equivalency with that of natural marshes [25, 28].

In the current study, we document rates of carbon sequestration in created *S*. *alterniflora* marshes in central North Carolina, USA, that range in age from 12 to 38 years. Two of the sites investigated here are transplanted fringing marshes that were created to function as living shorelines. The other three sites pre-date the living shoreline concept but were established by planting *S*. *alterniflora* in a sandy substrate, and occur in the same small geographic area. Consequently, we consider these sites to be representative of local living shoreline conditions. The defining feature of these sites is that all five were created by planting *S*. *alterniflora* in sandy sediments with negligible organic matter concentrations. As the marshes have matured, they have presumably increased in elevation (to keep pace with rising sea levels) and the coarse sandy sediment in which they were planted has been amended with inputs from plant belowground biomass, associated benthic and interstitial community production, and particulate matter trapped from incoming tidal waters. The sand background against which we are able to measure changes makes these sites model systems for the unequivocal determination of carbon sequestration rate. To our knowledge, the data presented here are the first published account of blue carbon sequestration in living shorelines.

## Methods

### Study Sites

**Fig 1:**
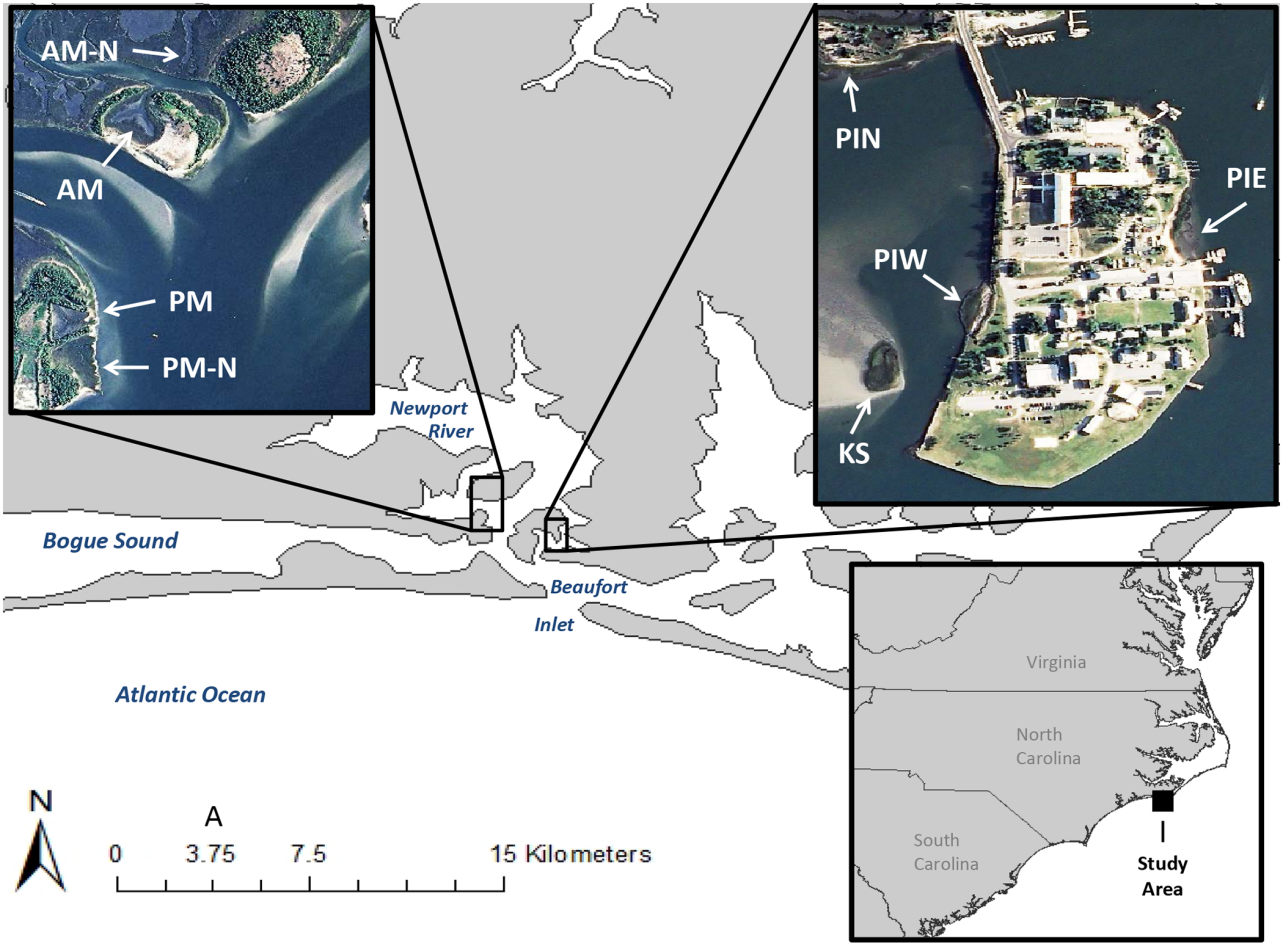
Sampling Locations. Samples were collected from PIE, PIW and PIN in 2012 and 2013 for analysis of belowground biomass/elevation trends. Cores were collected in 2014 from all sites except PIN for analysis of soil carbon.

The marshes investigated here include 3 natural (Pivers Island North [PIN], Army Marsh Natural [AM-N] and Port Marsh Natural [PM-N]), and 5 transplanted tidal salt marshes, all of which occur within a short distance of each other in the Newport River Estuary, NC (34°43′32″ N, 76°41′10″ W) (Fig 1; Table 1). Two of the transplanted sites, (Pivers Island West [PIW] and Pivers Island East [PIE]) were created as living shoreline projects on Pivers Island (PI). PIW, established in 2002, is a narrow created marsh bordered by a stone sill which was placed just offshore of the marsh to reduce wave energy. PIE was an eroding sandy beach planted with *Spartina alterniflora* (2000) and later amended with oyster shell placed just offshore of the marsh to serve as a point of attachment for oyster spat. This mound of oyster shell has since developed into a live reef. PIN is a nearby natural marsh that has been used as a reference site for both PIW and PIE [24]. The Kirby-Smith island site (KS) began as a small number (< 20) of *S*. *alterniflora* stems planted on an exposed tidal flat (Kirby-Smith, personal communication) and has since flourished into a 3200 m^2^ marsh. Both Port Marsh (PM) and Army Marsh (AM) were established on dredge spoil islands as part of mitigation actions [29, 30]. Because of their close proximity, all sites are assumed to experience similar water quality, weather, and climate conditions. All sites experience a mean tidal range of 1 m and all marshes are dominated by *S*. *alterniflora* [31]. The approximate areal expanse of each marsh was estimated with ArcMap 10.2.1 (Environmental Systems Research Institute, Redlands, CA) using National Agriculture Imagery Program aerial imagery (1 m resolution) that is available in the software’s world imagery base map layer.

**Table 1:** Sampling Sites.

Site	Year Planted	Total Area (m^2^)
**Kirby-Smith Island (KS)**	1979	3,200
**Port Marsh (PM)**	1990	12,800
**Port Marsh Natural (PM-N)**	-	24,000
**Army Marsh (AM)**	1995	17,300
**Army Marsh Natural (AM-N)**	-	850,000
**Pivers Island East (PIE)**	2000	250
**Pivers Island West (PIW)**	2002	200
**Pivers Island North (PIN)**	-	1200

Year planted and total areal expanse of each marsh (as of 2014) estimated from aerial photography.

### Sampling

#### Biomass

Carbon sequestration in tidal marshes occurs when the production of belowground root and rhizome material outpaces remineralization. Understanding patterns of belowground production and turnover is therefore essential to evaluating the capacity of a marsh to sequester carbon. In 2012, sediment cores (15 cm diameter x 30 cm deep) were collected for analysis of belowground biomass from each of the Pivers Island (PI) marshes (PIE, PIN, and PIW). At each site, samples were collected along three transects running perpendicular to the shoreline with one at low elevation, 0 m from the shoreline, and a second at high elevation (15 m inland) on each transect. Both low and high elevation cores were collected within the zone occupied by *S*. *alterniflora*. The elevation of low marsh cores ranged between -0.207 and -0.363 and averaged -0.288 cm North American Vertical Datum of 1988 (NAVD 88) across all sites, while high marsh core elevations ranged between 0.0114 and 0.345 and averaged 0.176 cm NAVD 88. Cores were rinsed over a 2 mm mesh sieve to remove sediment and shell, and the fraction retained on the mesh, defined as belowground biomass (BGB), was dried to a constant weight at 60°C. Dried samples were combusted in an ashing oven at 450°C for 4 hours and then reweighed. Organic matter content was determined from loss on ignition.

The holes created by core removal were filled with mesh bags containing a 50:50 mixture of sand and peat. These root ingrowth bags remained in place for one year, then were removed in 2013 and processed as described above. The material harvested from the bags after one year of growth was considered to be the net annual production of BGB. The mesh bags used for the ingrowth experiments did not have rigid frames and despite the fact that all remained buried and no surface erosion occurred, tidal motions resulted in a subsurface shifting of their proportions over time. Upon removal, several ingrowth bags had become wider and shallower than the original holes they were intended to fill. At the three most impacted sites, this resulted in a final sample depth of 10–12 cm. Sample depths in the 15 remaining bags ranged between 19 and 29 cm total. To facilitate among-site comparisons, the proportions of each bag were measured upon extraction and all were sliced into 0–10 cm and > 10 cm depth intervals for processing. Standing aboveground biomass (AGB) within the area of each core or ingrowth bag was clipped at the sediment surface, washed to remove epiphytes, dried to a constant weight, and combusted for determination of organic matter content as ash free dry weight. Because BGB in *S*. *alterniflora* has been shown to exhibit a great deal of temporal variability [32, 33], we sampled during the period of peak above ground biomass (late July) and all cores were collected within the same week.

#### Bulk Density and Carbon Content

In 2014, sediment cores (7 cm diameter x 35 cm deep) were collected from all sites except PIN for analysis of total sediment organic matter (this includes organic matter that is recognizable as BGB, as well as that which is small enough to pass the 2 mm sieve). Cores were collected from multiple elevations at each created marsh (2 each at KS, AM and PM, and 3 each at PIE and PIW) and from a single elevation at the natural reference marshes PM-N and AM-N. Precise locations were arbitrarily selected by tossing a 0.0625 m^2^ quadrat on the marsh surface in an area visually determined to have representative AGB coverage for that elevation. The total number of *S*. *alterniflora* stems within the quadrat was counted and the lengths of 10 randomly selected stems were measured. This data was used to estimate total standing AGB by a previously determined empirical relationship based on height/weight regression (S 1). Sediment cores were collected from within the area of each quadrat by inserting a PVC core tube in the ground to a depth of 40 cm. Due to the sandy nature of the substrate it was necessary to hammer the core tubes in with a rubber mallet at most sites. At the low elevation site at Port Marsh (PM-L), hammering resulted in ~5 cm of compaction. No noticeable compaction occurred in any of the other cores.

Cores were brought back to the lab and extruded in 5 cm increments. Each 5 cm section was dried at 60°C for 48 hours, weighed, and homogenized before a subsample was removed for elemental analysis. The remaining material was analyzed for organic matter content using the loss on ignition approach as described above. The subsamples were ground to a fine powder, weighed into silver capsules, acidified with 3N HCl (by drop-wise addition until bubbling stopped) to remove carbonates and then dried at 50°C. Carbon (C) and nitrogen (N) content were determined with a Costech ECS 4010 elemental analyzer. The average standard deviation of triplicate samples was 0.05% C and 0.004% N. Bulk density (dry weight cm^-3^) and carbon density (bulk density * % organic C content) were calculated for each 5 cm interval.

At all transplanted sites, initial marsh establishment occurred via the planting of *S*. *alterniflora* in a sandy intertidal substrate that was not colonized by emergent macrophytes prior to planting. We used 35 cm deep cores with the goal of collecting sediments that were deep enough to have had no input from surface vegetation and, thus, represent time = 0 sediment conditions. At all sites (other than the natural marshes) a coarse grey sand layer was present and extended from depths of 15–20 cm to the bottom of the core. This deep layer was devoid of identifiable root material and was visually distinct from the brown, silty upper regions of the cores. This deep sand layer is, therefore, assumed to represent initial or background conditions for each marsh. Background carbon content (the amount present in the 30–35 cm depth interval) was subtracted from each of the 5 cm intervals. Total carbon stocks in the top 30 cm were determined by summing the background-corrected values for each interval up to 30 cm and annual carbon sequestration was determined by dividing carbon stock by marsh age. This approach likely results in conservative estimates of carbon sequestration, as background-corrections are made for newly deposited sediments as well as the original underlying sediment. All sampling occurred below Mean High Water (established via the National Tidal Datum Epoch, 19 yr. average), and was therefore in state waters. No special permission is needed to collect plants and sediments in these areas for research purposes. There were no endangered or protected species collected, or impacted by, the sampling effort.

#### Elevation

Marsh sediment surface elevations (NAVD 88) were determined by laser leveling to a local benchmark for all sampling sites other than PM and AM. The benchmark used for KS and PIW was a Surface Elevation Table (SET) benchmark installed within the PIW living shoreline. The SET benchmark elevation was determined using the National Geodetic Survey Online Positioning User System (OPUS). The accuracy of elevation determinations from the SET benchmark was determined to be (+/- 7cm). For PIE a nearby benchmark, National Center for Coastal and Ocean Science (NCCOS) Beaufort Commemorative Mark (PID DG9239), was used for reference. The elevation at the PM and AM marshes was determined by Real Time Kinematic GPS with the base station located and referenced to bench mark 865 6483 E TIDAL (PID DE7961). The accuracy of these elevations was +/- 6.6 cm.

#### Statistics

Significant differences in BGB between high and low elevation marsh cores were analyzed via Wilcoxon-Signed Rank tests after Shapiro-Wilks test for normality (and subsequent attempts at data transformation) indicated a non-normal distribution in BGB data from low elevation cores. All statistical analyses were conducted using JMP software. (JMP 11.2.1, SAS institute Inc.).

## Results

The five transplanted marshes included in this study range in age from 12 to 38 years and vary widely in size (Table 1). In the time since planting, all marshes have expanded in areal extent into regions that were not planted when the marsh was initially created. As a result, the precise time span represented is not known for all cores. This is particularly true of the low elevation cores collected from PIE, PIW and the core on the advancing edge of the marsh at KS. For age related comparisons, we used core data from sites that are known to have been planted at the time of marsh creation and that thus, represent the full age of each site. These cores are hereafter referred to as the cores of known age.

### Biomass

**Fig 2:**
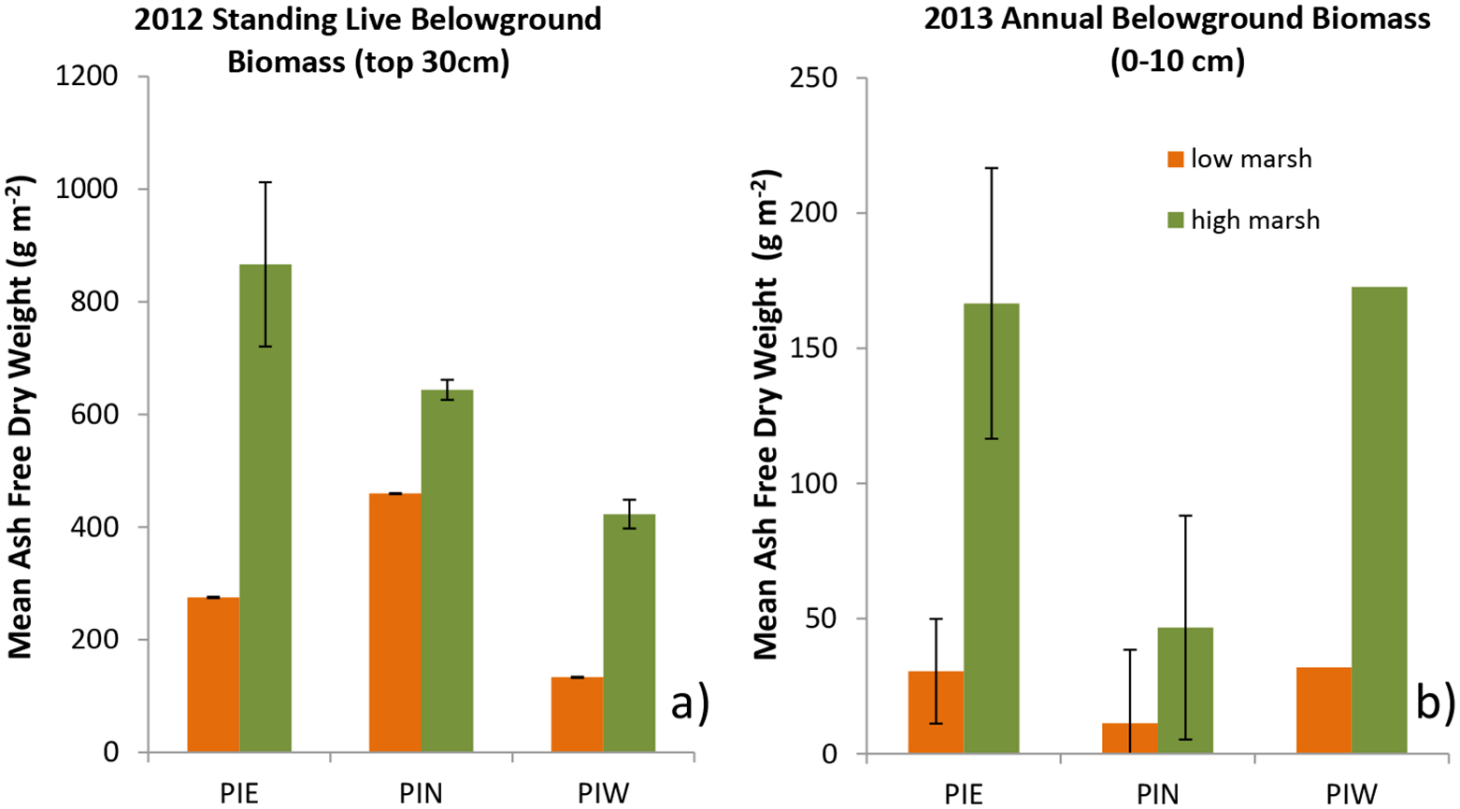
Belowground biomass. a) Total belowground biomass (> 2 mm) in 15 x 30 cm core, b) Total biomass in ingrowth bags after one year of growth. Due to changes in shape of bags overtime, only the top 10 cm is used for comparison.

**Fig 3:**
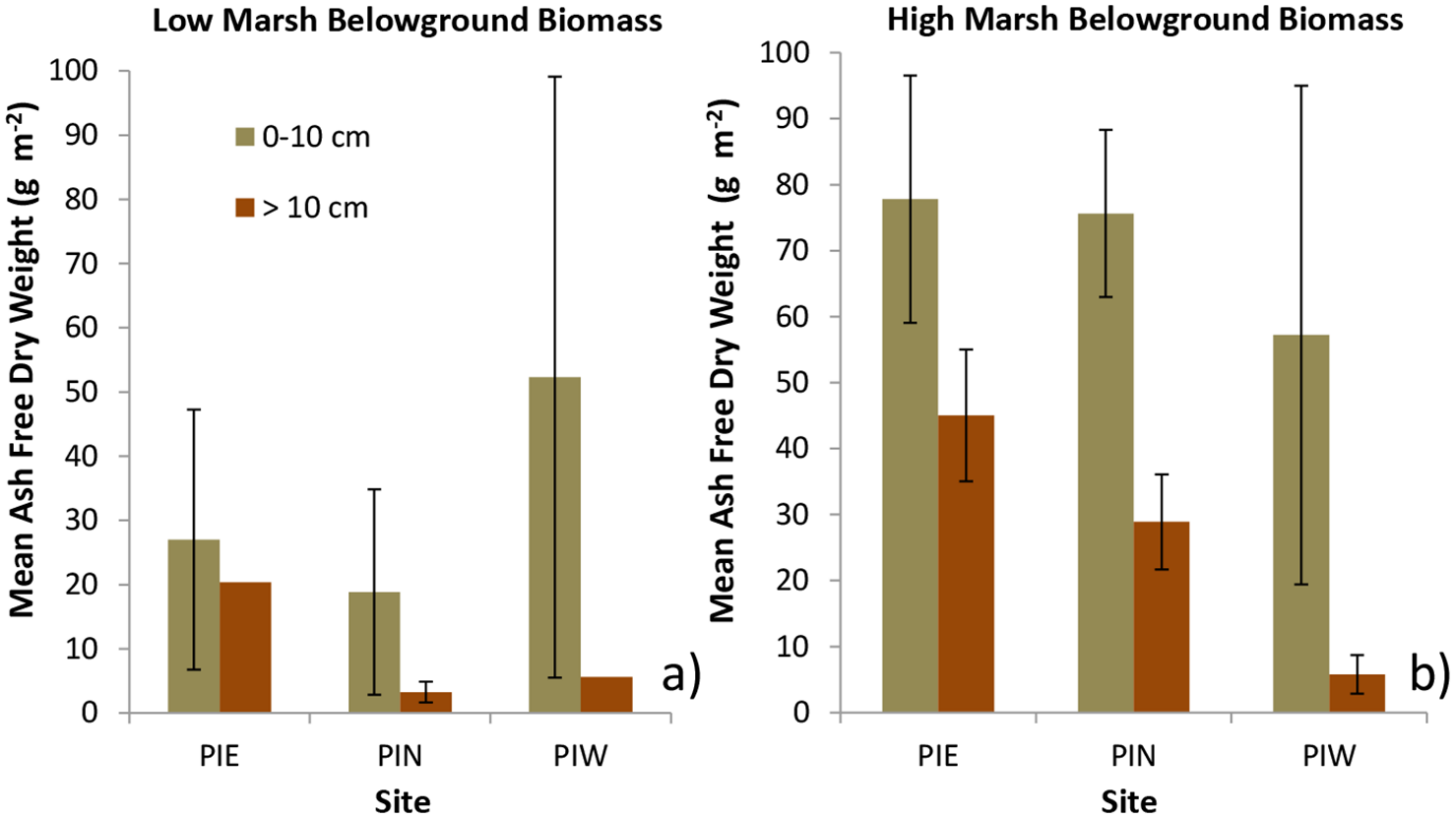
Belowground biomass production by depth. Total amount of belowground biomass (> 2 mm) by depth interval in ingrowth bags after one year of growth in: a) low, and b) high elevation cores. Cores were 10–30 cm in total depth.

Statistical analysis of standing belowground biomass collected from low and high elevation regions of PI marshes in 2012 indicated a positive relationship between BGB and relative elevation (P = 0.04; Fig 2a). Aboveground biomass ranged between 150 and 1588 g m^-2^ and was not correlated with BGB. The trend of increasing BGB with elevation was also apparent in the root ingrowth bags where annual BGB production in the high marsh sites was up to 4 times that of low marsh sites (P < 0.01; Fig 2b). Analysis of biomass distribution by depth in ingrowth bags indicated that the majority of annual BGB production was found in the upper 10 cm of the soil; however, substantial belowground production occurred at depths greater than 10 cm, particularly in the high elevation plots (Fig 3a and 3b).

### Bulk Density and Carbon Content

At all transplanted marsh sites other than PM-L, percent organic matter (OM) was < 1% below a depth of 20 cm. The deep organic matter (OM) content of PM-L (1.8%) was slightly higher than other transplanted marsh cores. Carbon densities in surface sediments (top 5 cm) ranged between 0.004 and 0.06 g C cm^- 3^ (Table 2) and were not correlated with marsh age. Total carbon stocks in the top 30 cm of each core ranged between 0.7 and 7.7 kg C m^-2^ with the highest values observed in the natural marshes (Table 2). Carbon sequestration rates, calculated by dividing the carbon stock by marsh age, ranged between 58 and 283 g C m^-2^ yr^-1^ with the highest sequestration rates occurring in the youngest marshes (Table 2).

**Table 2:** Elevation and carbon characteristics of individual cores collected in 2014.

SITE	Elevation m (NAVD 88)	AbovegroundBiomass	C density 0–5 cm	Total C stock 0-30cm	Measured sequestration rate
		(g m^-2^)	(g C cm^-3^)	(kg C m^-2^)	(g C m^-2^ yr^-1^)
**KS-H**	**-0.14**	**2164**	**0.024**	**2.76**	**73**
KS-L	**-0.232**	541	**0.014**	**1.10**	208.6†
**PM-H**	**0.09**	**215**	**0.02**	**2.85**	**119**
**PM-L**	**-0.3**	**453**	**0.011**	**2.96**	**123**
PM-N	**-0.1**	697	**0.019**	**5.65**	*
**AM-H**	**0.06**	**274**	**0.023**	**2.37**	**125**
**AM-L**	**-0.14**	**173**	**0.015**	**1.63**	**86**
AM-N	**0.08**	293	**0.026**	**9.86**	*
PIE-H	**0.29**	524	-	-	**
**PIE-M**	**0.004**	**662**	**0.009**	**2.92**	**208**
PIE-L	**-0.22**	216	**0.014**	**1.46**	104†
**PIW-H**	**0.19**	**57**	**0.016**	**2.51**	**209**
**PIW-M**	**-0.07**	**733**	**0.06**	**3.39**	**283**
PIW-L	**-0.3**	72	**0.009**	**0.70**	58†

Bold font represents cores that were collected from regions that were known to be planted at the time of marsh creation (cores of known age). Sites are designated as high (H), mid (M), or low (L) based on their relative elevation within each site. N = natural reference marshes. Values of carbon density are reported for the top 5 cm of each core. Total C stock was calculated as the sum of bulk density times % organic matter for each 5 cm interval. Sequestration rate was calculated as total C stock divided by marsh age.

* Marsh age not determined.

** Core only extended to 20 cm depth.

^†^ These sites have not been colonized by *S*. *alterniflora* for entire duration of marsh age, thus sequestration rates may be underestimated.

**Fig 4:**
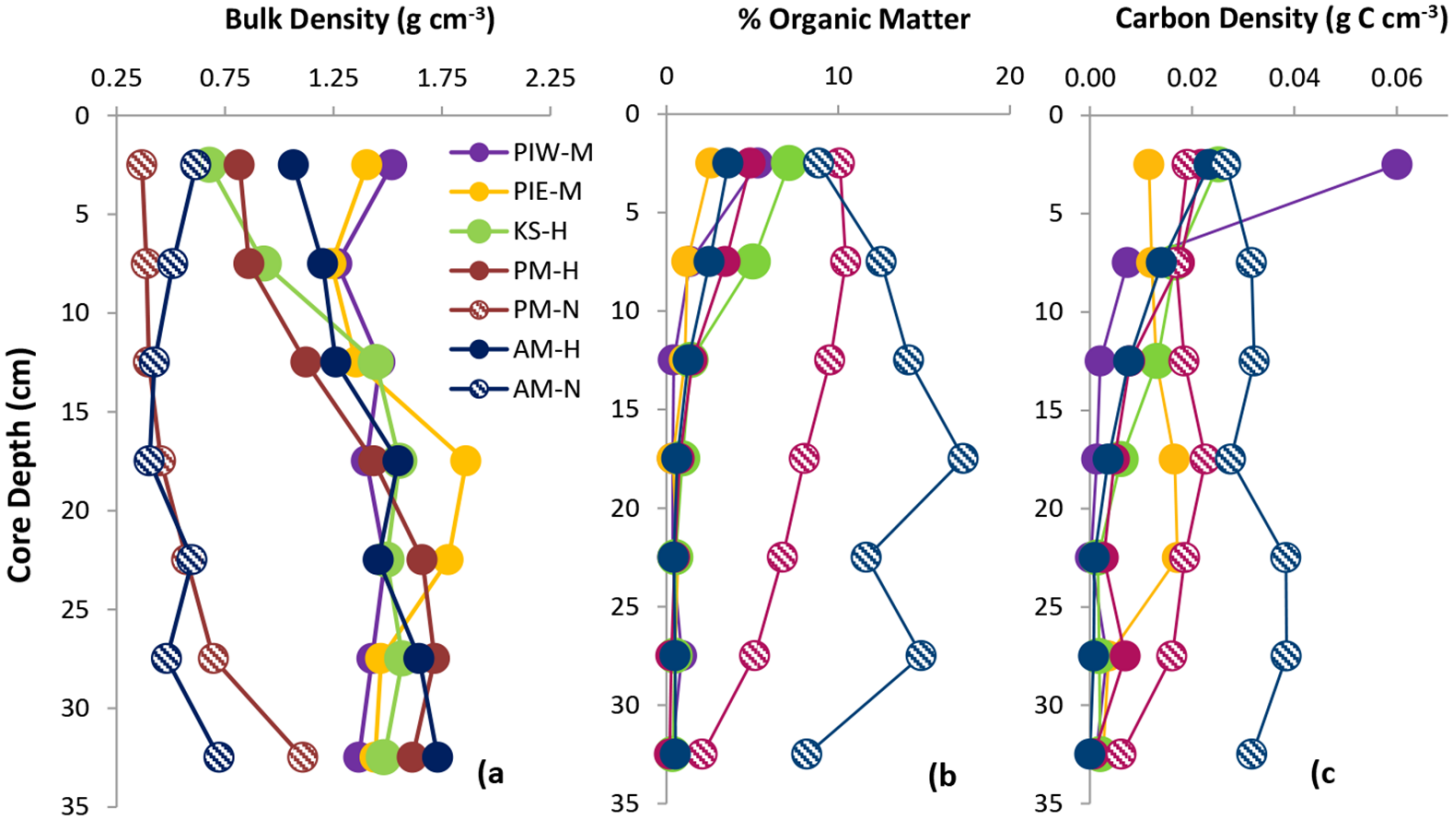
Depth profiles. Comparisons of: a) soil bulk density, b) soil percent organic matter, and c) soil carbon density in core of known age from each site that is closest to 0 m NAVD88 elevation.

Comparisons of down-core profiles of bulk density, percent organic matter and carbon density among sites clearly illustrate the carbon footprint of marsh vegetation in the surface sediments of these marshes (Fig 4a–4c). Bulk density of surface sediments ranged from 0.36–1.56 g cm^-3^ with the lowest values detected in the natural marshes (Fig 4a). Bulk density values were relatively uniform across the depth profile in natural marshes, but increased with depth at the created marshes. Natural marshes contained greater amounts of organic matter than transplanted marshes throughout the core depth profile, although the difference was greatest at depths below 10 cm (Fig 4b). Carbon density profiles displayed a similar pattern with elevated values in the upper 10 cm of transplanted marshes and more uniform values in the natural marshes (Fig 4c). Elevated bulk density and % OM in surface sediments at PIW resulted in a carbon density value that was ~3 times greater than at any of the other sites. There was a large amount of variability in carbon density with depth among cores from the same marsh but no consistent trends with elevation or distance from the water’s edge. No detectable relationship occurred between carbon density and the amount of above ground biomass, elevation, marsh age, or sediment carbon stock.

**Fig 5:**
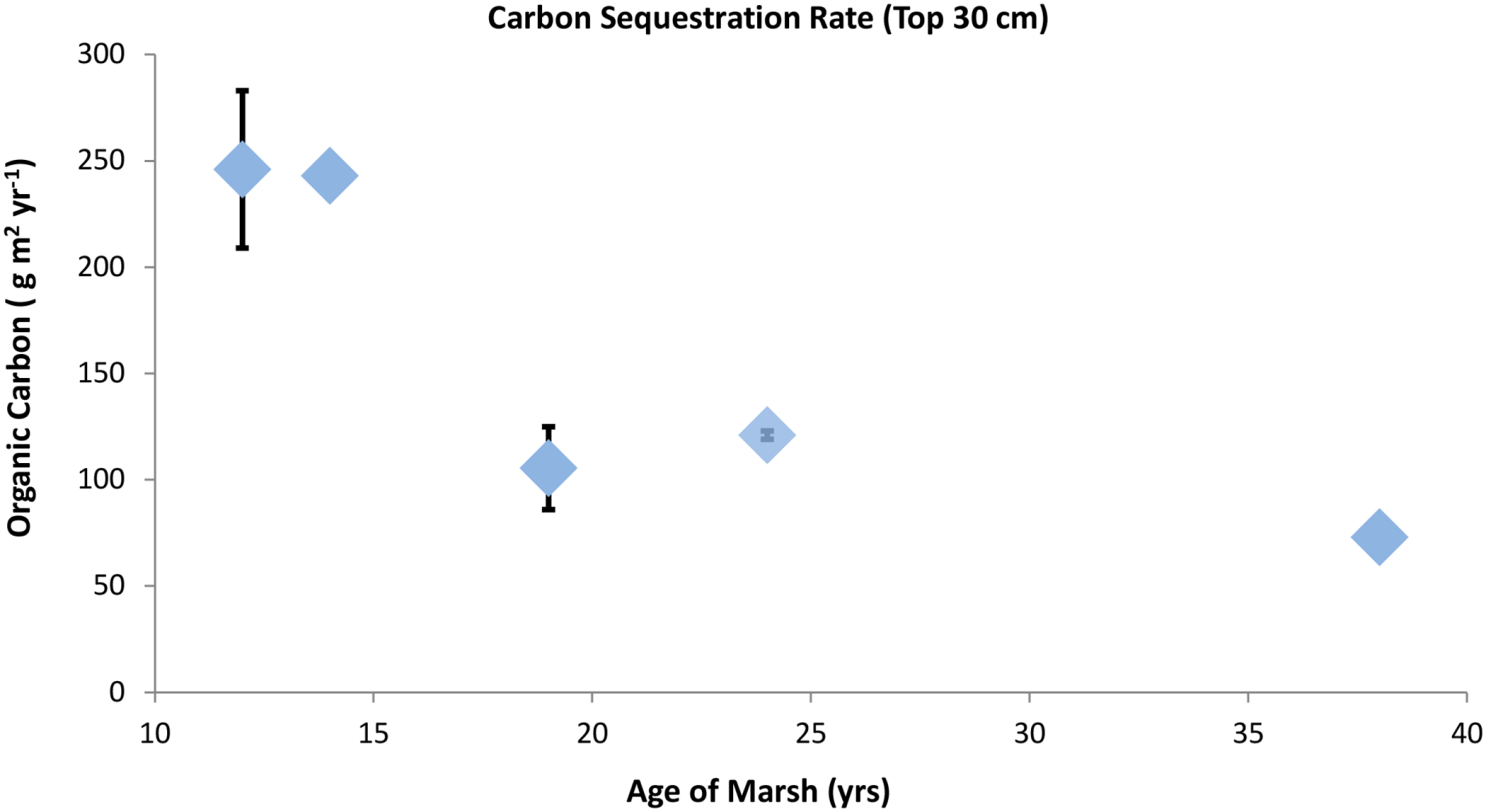
Carbon sequestration rate. Rates were calculated as: (total carbon stock—background)/marsh age, for cores of known age from each marsh. Error bars show maximum and minimum values from replicate cores from each site. Points without error bars (13, and 38 yrs.) represent single cores.

**Fig 6:**
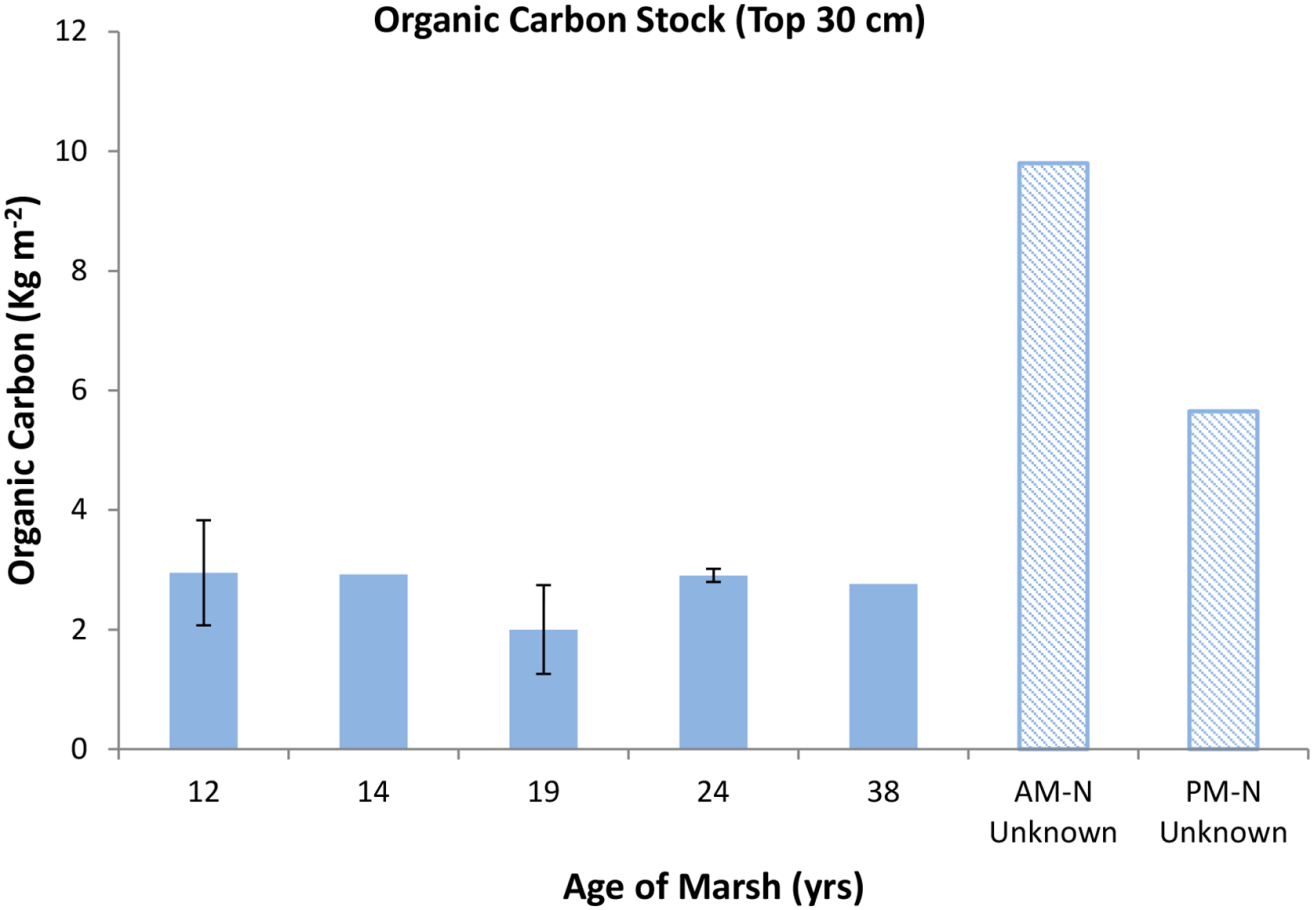
Total carbon stock by marsh age. Data represent averages of total organic carbon (0–30 cm), from cores of known age. Error bars show maximum and minimum values from replicate cores. Points without error bars (13, and 38 yrs., AM-N and PM-N) represent single cores.

Annual carbon sequestration rates in the cores of known age ranged from 72 to 283 g C m^-2^. Sequestration rate decreased with marsh age with those sites younger than 15 years exhibiting rates 2 to 3 times greater than older sites (Fig 5). However, comparison of total carbon stocks by marsh age in these same cores did not suggest a parallel relationship between C stock and age. Instead, the younger marshes contained either more, or equivalent amounts of buried carbon when compared to the 24 and 38 year old sites (Fig 6).

**Fig 7:**
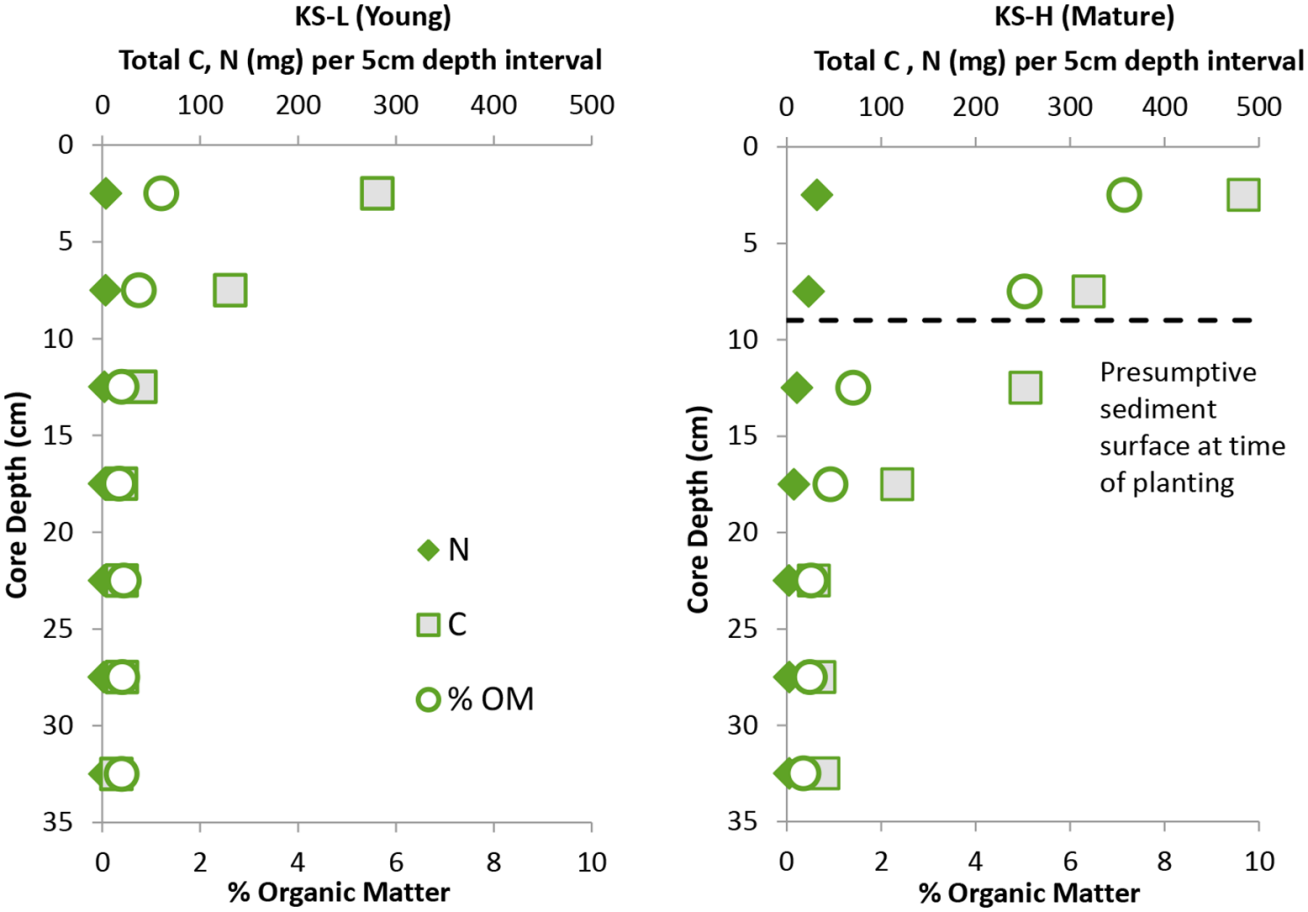
Organic matter, carbon and nitrogen profiles. Cores were collected at mature (~ 38 yr. old) and young (< 5 yr. old) regions of the same marsh. The presumptive sediment surface at time of planting was calculated by assuming a rate of surface elevation increase equivalent to the locally measured rate of sea level rise (see text for details).

Comparison of a recently colonized region of the KS marsh (KS-L; young) with a more mature region of the same marsh (KS-H; mature) illustrates temporal changes in sediment properties that result from *S*. *alterniflora* colonization (Fig 7). Analysis of aerial photography indicates the KS marsh has increased in area over the past two decades. The KS young core was collected on the advancing edge of the marsh. This site is characterized by sparse, low growing *S*. *alterniflora (*average stem height = 56 cm, estimated biomass = 541 g m^-2^ *)* and coarse sandy sediments. The precise amount of time that this region has been colonized is not known, but we estimate it to be 5 years or less. The KS mature core site represents the oldest region of the marsh and is characterized by denser and taller *S*. *alterniflora* (average stem height = 107 cm, estimated biomass = 2164 g m^-2^) and sediments with a greater organic content (Table 2; Fig 7). Data from the two cores indicates elevated N content in the surface layers of the mature marsh and elevated C in the mature relative to young marsh core at all depths.

One of the mechanisms by which coastal marshes keep pace with sea level rise (SLR) is through the deposition of materials that are carried onto the marsh platform during flood tides [34, 35]. While the rates of deposition may vary from low to high regions of the same marsh, as a function of variable inundation time, the rate of local SLR has been shown to provide a reasonable approximation of marsh elevation change over time [36]. The average rate of SLR in central North Carolina over the 60 year period between 1953 and 2013 is ~2.7 mm yr^-1^ [37]. Pb-210 and Cs-137 based accretion rates from marshes in our study region average 2.3 mm yr^-1^ [28]. Using these two rates as high and low estimates of marsh elevation change, we hypothesize that 38 years ago when this site was initially planted, the sediment surface of the KS high site was somewhere between 8 and10 cm lower than it is today. Our laser leveling data indicate that the young site is currently 9.2 cm lower in elevation than the mature site (Table 2). The portion of the mature marsh profile that falls below the presumptive initial sediment surface is strikingly similar to the young marsh profile, e.g., the 10–15 cm segment from the mature marsh resembles the 0–5 cm section of the young marsh in terms of C and N abundance.

### Elevation

**Fig 8:**
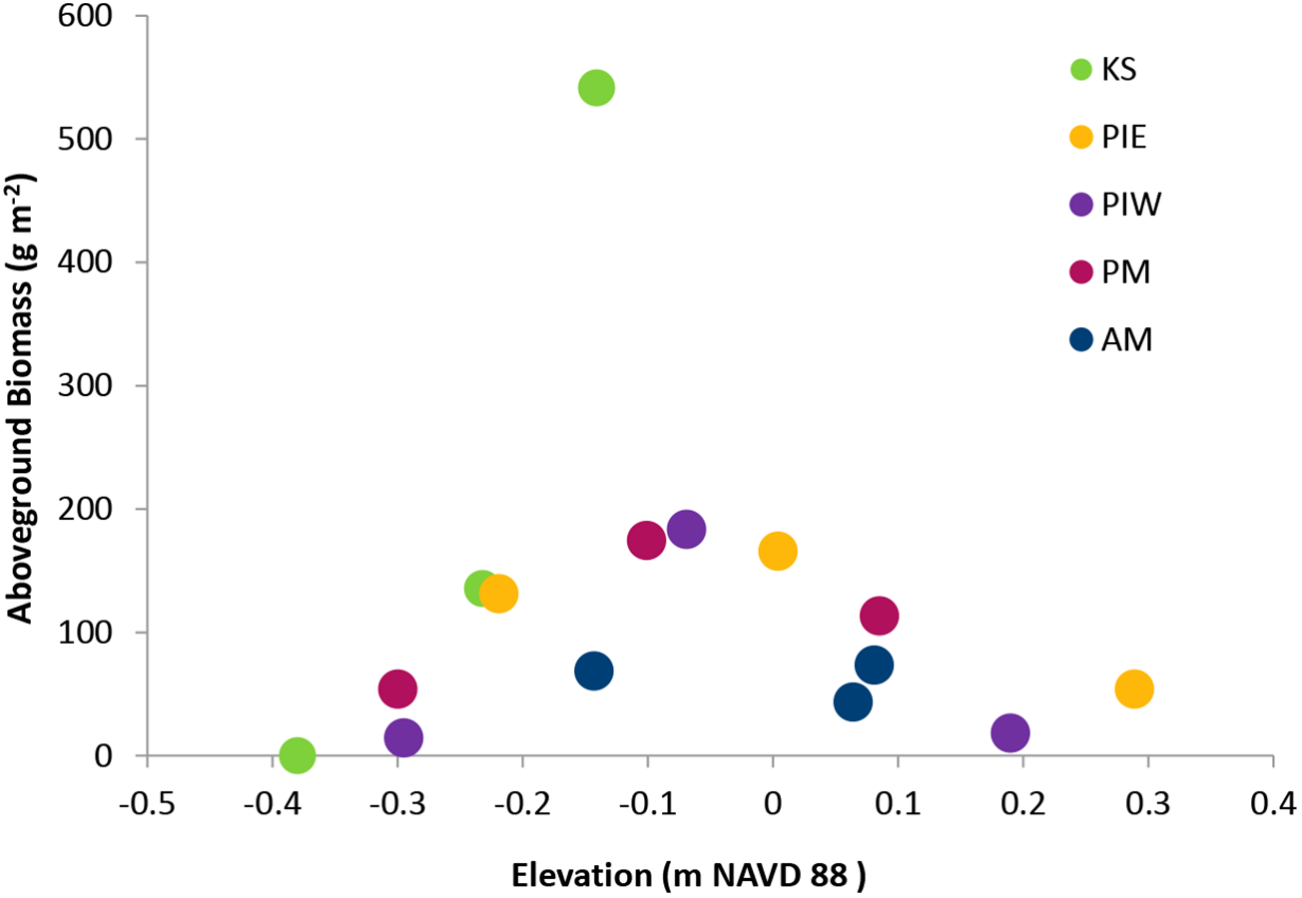
Live aboveground biomass by elevation. Total aboveground biomass measured at the collection site of each soil core.

The habitat requirements of *S*. *alterniflora* dictate that all sites sampled here fall within the intertidal zone. The elevations of our core sites ranged from -38 to +29 cm NAVD 88. The living shoreline sites (PIE and PIW) had the greatest slopes, with ~50 cm of elevation gain from shoreline to upland marsh border. The KS site was lower than the rest, with the highest part of the marsh at an elevation of -0.14 m NAVD 88. Aboveground biomass, compared across all sites, exhibited a parabolic relationship with elevation, with peak biomass occurring around -0.1 m NAVD 88 (Fig 8). There were no detectable relationships between elevation and either carbon density or carbon sequestration rate.

## Discussion

### Belowground Biomass

The results of these experiments demonstrated greater belowground biomass production in plots that were 15 m inland (and consequently at higher elevation) than plots at the shoreline (Fig 2). The marshes in which the ingrowth root bag experiments were conducted are narrow fringing marshes with relatively steep slopes (~50 cm of elevation change over 20–30 m distance). Earlier studies have found increases in both belowground biomass and belowground production with elevation [38–40]. Given the positive correlation between belowground biomass and elevation, one might predict greater rates of carbon sequestration at higher marsh elevations. The data collected in this study, however, failed to show a consistent relationship between elevation and carbon sequestration rate, possibly due to lack of a consistent relationship between created marsh age and elevation (Table 2). This lack of an association between carbon sequestration and elevation, despite up to 4 times greater BGB production in the high marsh, may also result from greater rates of BGB remineralization in high vs. low elevation marsh sediments. Greater high marsh decomposition rates make intuitive sense in these sandy, well-flushed substrates where higher elevation marsh sediments likely experience greater oxygen penetration and, therefore, higher decomposition rates. Prior analyses of the relationship between decomposition and inundation have been mixed, with some detecting an inverse relationship between soil respiration and inundation time [41] while others found no relationship between flooding frequency and decomposition rates [39, 42].

### Bulk Density and Carbon Content

Soil structure and composition have been shown to be indicative of the maturity of wetland soils and are therefore useful proxies of the extent to which created wetlands are functionally equivalent to natural wetlands [28, 43]. It has been suggested that at a minimum, multiple decades are necessary for the structural properties of created marsh soils to mirror those of natural marshes [25, 44]. Natural wetlands are characterized by lower bulk density and greater organic matter content than their created counterparts [26, 45–47]. In typical created wetlands, bulk density decreases over time as a result of the accumulation of both living and dead root and rhizome material. This decrease in density occurs in concert with increases in organic matter concentration. In the current study, created marshes greater than 20 years in age (KS and PM) were most similar to natural marshes in terms of bulk density but only in the top 5–10 cm (Fig 4a). While older created marshes had greater OM content in the upper 5 cm than younger created marshes, they still lagged behind the natural marshes in OM content by 25–40%. Because all of the marshes in this study are exposed to the same environmental conditions, exist at similar tidal elevations, and are dominated by *S*. *alterniflora*, we hypothesize that natural marshes represent an “endpoint” that created sites will reach given enough time.

### Carbon Sequestration

Among the properties that contribute to the carbon sequestration capacity of coastal wetlands is their ability to increase in elevation in response to sea level rise [48]. As this increase occurs, the zone of marsh soil that has been enriched with carbon from belowground biomass becomes thicker. This point is illustrated in the carbon profiles of the young and mature marsh sites at KS (Fig 7). The active root zone in these marshes extends to ~15 cm below the surface. Thus C that is being produced today will be located in the upper 15 cm. As the marsh grows in elevation, the deeper part of this zone will no longer receive inputs of new material. If we assume the marsh at KS has increased in elevation on pace with sea level rise, then at the time of planting, the marsh surface would have been 8–10 cm lower than it is today. This is consistent with the carbon profile, as the carbon enriched region extends roughly 15 cm below the presumptive surface at the time of planting. Vertical carbon density profiles of natural marshes are the product of decades worth of belowground biomass production and elevation increase that have allowed the soils to come to an “equilibrium” with respect to carbon inputs and remineralization [49]. In newly created marshes, we would expect an increase in soil % C until the point that this equilibrium is reached.

In natural marshes, sediment ages are generally determined by inferring long-term sediment accretion rates from radio-isotope profiles [50, 51]. However, radio-isotope profiles can be difficult to interpret, particularly in sandy sediments that are readily reshuffled by tidal motions and bioturbation. The precise ages of all created marshes investigated here are known, and the background sediment conditions are distinct from the overlying marsh sediment giving us the luxury of not having to rely on proxies of sediment age. As a result, we have confidence in our carbon sequestration rate determinations which range from 58 to 283 g C m^-2^ yr^-1^ and decrease with increasing marsh age (Fig 5). Published rates of carbon sequestration from a range of tidal saline wetlands vary from 21 to 1713 g C m^-2^ yr^-1^, with averages of 22 to 244 g C m^-2^ yr^-1^, thus the rates determined here fall within the range of published values [22, 23, 52]. Our data suggest that the wide range of previously published values for marsh carbon sequestration rate may be, in part, due to inclusion of sites with varying ages/stages of maturity.

**Fig 9:**
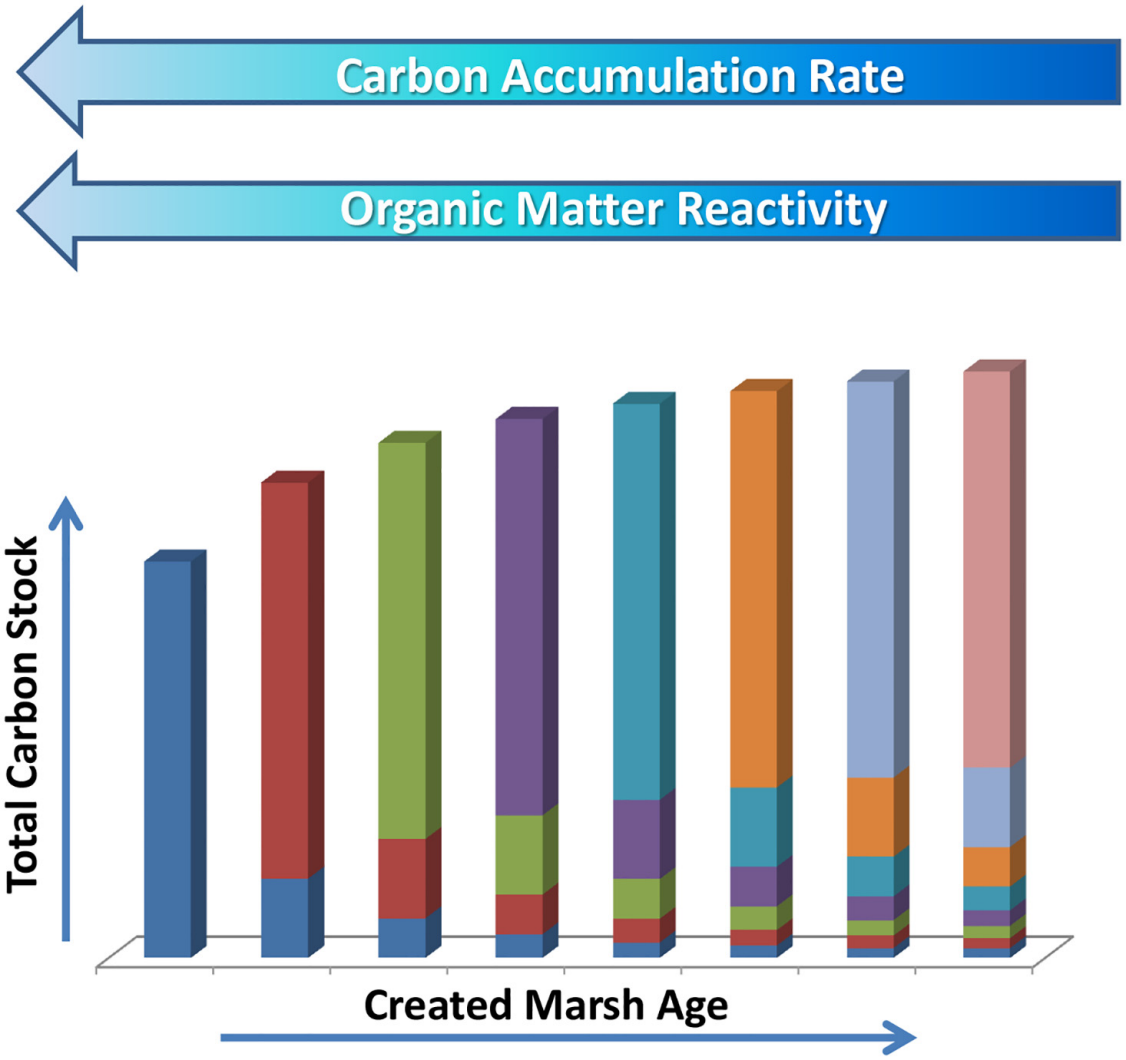
Conceptual model of carbon burial and turnover in a newly created marsh. At each time step a new “cohort” of carbon is added to soil as BGB. Each new cohort is represented by a different color. The decrease in size of a given cohort over time represents remineralization of the labile and semi-labile fractions. This remineralization continues until only the recalcitrant material remains. The result is that over time the bulk reactivity of the soil decreases as does the time-averaged carbon sequestration rate. Note that in this conceptual model, the amount of new carbon being input each year is constant. In a natural marsh, biomass, and therefore new carbon inputs, will fluctuate annually. As a result carbon stock is likely to fluctuate over time but will show a general upward trend over long time scales.

In created marshes, like those investigated here, initial carbon accumulation rates provide exaggerated estimates of long-term sequestration. This is a result of planting carbon rich vegetation in a carbon poor, sandy substrate. As the plants mature, the roots die and begin to decay but not all of this material decays on the same time scale. The highly reactive fraction may disappear within days while a portion will be preserved over centennial to millennial timescales [53, 54]. Ultimately only a small fraction of the original material is refractory enough to be preserved for long time periods and contribute to the soil carbon pool. Thus as a soil ages, the relative contribution of labile and semi-labile components will decrease and, as a result, so will the apparent C sequestration rate (Fig 9) until an equilibrium between turnover and new inputs is reached.

An understanding that the apparent rate at which C is incorporated into soils decreases over time is critical to determinations of the long-term carbon sequestration potential of created marshes. Carbon which is incorporated into biomass but then respired to CO_2_ in the short term (years to decades) should not be quantified as part of the C sequestration term. Defining the appropriate sequestration rate, however, is complicated by the fact that most created marshes are less than 50 years old. In the created marshes examined here, the rate of carbon sequestration appears to decrease precipitously between ~ 13 and 20 years (Fig 5). After this point, the rate appears to continue decreasing, though much less dramatically. We hypothesize that these older marshes are approaching the equilibrium between production and remineralization. This interpretation is supported by the finding that organic matter in constructed marshes < 20 years in age in this same area is more labile than the OM at older sites [28]. Despite the lower % OM in young sites, these authors noted greater C-normalized rates of heterotrophic metabolism due to the greater bioavailability of OM in younger sites.

Previously published rates of carbon sequestration in tidal salt marshes suggest a global average value on the order of 210 to 244 g C m^-2^ [22, 23]. These averages were calculated using the carbon accumulation rate approach in which sediment accumulation rates are multiplied by surface carbon densities. Surface sediments are younger, and therefore enriched in carbon, as a result, the application of surface carbon density values to the entire core, can result in overestimates of carbon accumulation. The trend of decreasing carbon sequestration rates with increasing marsh age detected here demonstrates the potential bias associated with using only recent surface sediments to calculate carbon sequestration. We suggest that carbon accumulation and carbon sequestration are strongly related but not interchangeable.

### Implications for the Blue Carbon Potential of Living Shorelines

Living shorelines are installed to prevent shoreline erosion. By providing a barrier between the ocean and the eroding shoreline, they have the instantaneous carbon benefit of stopping erosion-related carbon losses along that initial shoreline [17]. As living shorelines grow to maturity they, unlike hardened shorelines, have the capacity to assimilate atmospheric carbon dioxide which may remain stored underground for centuries. In many cases, these sites are narrow (< 30 m wide) and small in linear extent. Often they are installed on a property by property basis thus, the impact of the individual living shoreline is small but as they continue to gain popularity as an erosion control strategy their cumulative impacts may become substantial.

Because the carbon in our older restored marshes appears to be reaching equilibrium with respect to organic matter fluxes, we suggest that 70 to 80 g C m^-2^ yr^-1^ is a reasonable estimate for the long term (100 year) blue carbon sequestration rate for fringing marsh living shorelines in North Carolina. These rates are on the lower end of the average carbon burial rates in *S*. *alterniflora* marshes [22, 23]. This is likely the result of the sandy, well flushed nature of these sites which leads to lower soil % OM content in natural fringing marshes than interior marshes ([24] and references therein). A previous analysis indicates that typical living shorelines in this region are 20–30 m in width [55]. There are currently 124 permitted shorelines in North Carolina, with a cumulative total length of 10 km, thus we estimate a cumulative area of 250,000 m^2^ of existing marsh-sill based living shoreline. At a sequestration rate of 75 g C m^-2^ yr^-1^ this area of living shoreline translates to a cumulative annual carbon benefit of 18.75 metric tons, equivalent to the removal of 64 metric tons of CO_2_. The U.S. Energy Information Administration suggests that each gallon of E10 gasoline (10% ethanol) burned results in the production of 18.95 lbs of CO_2_. Using these values, we estimate that the current sill-based living shorelines in North Carolina (roughly 6 miles in total length) offset the equivalent of 7525 gallons of gasoline consumption each year.

According to the North Carolina Division of Coastal Management, there are greater than 12,000 linear miles of estuarine shoreline in North Carolina. As of 2012, 839 of those miles were armored with some type of stabilization structure, more than half of them with bulkheads [56]. Coastal regions are experiencing greater rates of population growth than their inland counterparts [57], and with this population growth comes increased shoreline development and associated armoring [58, 59]. Given these trends, it is likely that we will see increased shoreline armoring in the future. If the living shoreline approach becomes a more standard practice, this could result in a substantial carbon benefit.

## Conclusions

The IPCC has estimated that global CO_2_ emissions must be decreased to less than 15% of their 2000 levels by 2050 if we are to avoid a global average temperature increase of 2°C. While an 85% reduction in emissions seems unattainable, it has been suggested that a combination of reduced emissions and increased CO_2_ sequestration may make this goal more reachable [60]. In low energy settings, living shorelines represent a more desirable erosion control solution than hardened structures. This desirability stems from the fact that unlike vertical bulkheads and rip-rap, living shoreline designs maintain a connection between upland and open water regions and thus provide habitat and water quality enhancement benefits commonly associated with natural salt marshes. In addition, living shorelines can be more resilient to SLR via sediment accretion [24] and can provide superior storm protection [11]. The data presented here suggest that carbon sequestration should be added to the list of services provided by living shorelines and that these structures can play a role in a global strategy of reducing atmospheric CO_2_ by increasing sequestration.

## Supporting Information

S1 File *Spartina alterniflora* height-weight regression. Description of how the height/weight regression used to estimate *Spartina alterniflora* biomass was generated.
(DOC)
