# Supplementary material for: Living Shorelines: Coastal Resilience with a Blue Carbon Benefit
Source: PLoS One. 2015 Nov 16;10(11):e0142595. doi: 10.1371/journal.pone.0142595 (PMC4646691; doi:10.1371/journal.pone.0142595)

**S.1 *Spartina alterniflora* height-weight regression.**

The relationship between total plant height and biomass was calculated using plants collected from four different marshes on Pivers Island, near Beaufort, North Carolina. Collections occurred in 2004, 2006 and 2007. At each marsh, 10-15 plants were randomly selected from plots located within 5 m of the shoreline (518 stems total). Total height of each stem was measured in the field, then stems were clipped at the sediment surface and returned to the laboratory where they were washed to remove sediment and epiphytes. Stems were dried at 60^0^C for 24 hours, and the dry weight of each stem was recorded and regressed against stem height. Data from all years were pooled to develop the following height weight regression:


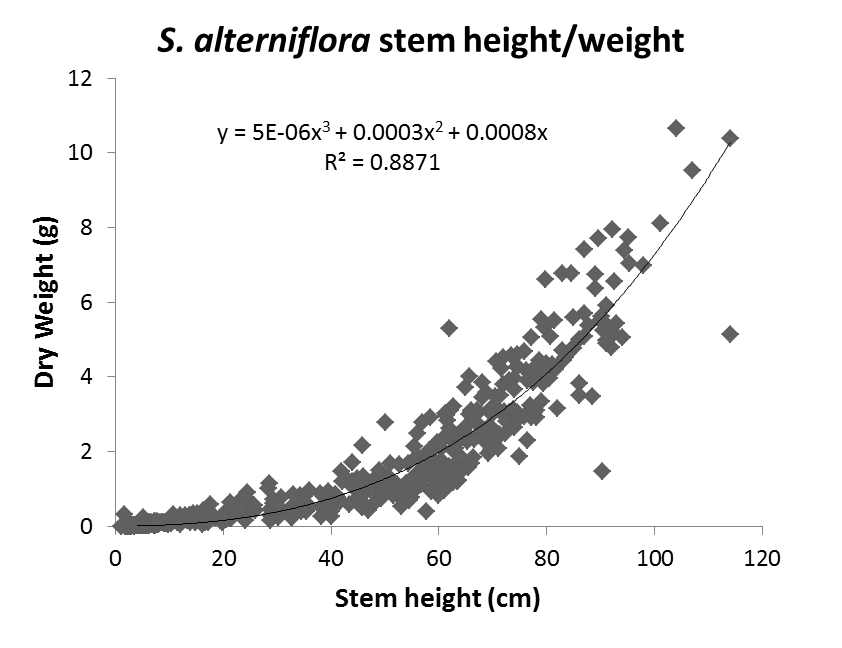

Supplement: S1 File — Description of how the height/weight regression used to estimate Spartina alterniflora biomass was generated. (DOC) [file pone.0142595.s001.doc]
